# Influence of Sampling Site and other Environmental Factors on the Bacterial Community Composition of Domestic Washing Machines

**DOI:** 10.3390/microorganisms8010030

**Published:** 2019-12-22

**Authors:** Susanne Jacksch, Dominik Kaiser, Severin Weis, Mirko Weide, Stefan Ratering, Sylvia Schnell, Markus Egert

**Affiliations:** 1 Faculty of Medical and Life Sciences, Institute of Precision Medicine, Microbiology and Hygiene Group, Furtwangen University, 78054 Villingen-Schwenningen, Germany; Susanne.Jacksch@hs-furtwangen.de (S.J.); dominik.kaiser87@gmail.com (D.K.); Severin.Weis@hs-furtwangen.de (S.W.); 2 International Research & Development—Laundry & Home Care, Henkel AG & Co. KGaA, 40191 Düsseldorf, Germany; mirko.weide@henkel.com; 3 Institute of Applied Microbiology, Research Centre for BioSystems, Land Use, and Nutrition (IFZ), Justus-Liebig-University Giessen, 35392 Giessen, Germany; stefan.ratering@umwelt.uni-giessen.de (S.R.); sylvia.schnell@umwelt.uni-giessen.de (S.S.)

**Keywords:** washing machines, bacterial diversity, biofilms, amplicon sequencing, hygiene

## Abstract

Modern, mainly sustainability-driven trends, such as low-temperature washing or bleach-free liquid detergents, facilitate microbial survival of the laundry processes. Favourable growth conditions like humidity, warmth and sufficient nutrients also contribute to microbial colonization of washing machines. Such colonization might lead to negatively perceived staining, corrosion of washing machine parts and surfaces, as well as machine and laundry malodour. In this study, we characterized the bacterial community of 13 domestic washing machines at four different sampling sites (detergent drawer, door seal, sump and fibres collected from the washing solution) using 16S rRNA gene pyrosequencing and statistically analysed associations with environmental and user-dependent factors. Across 50 investigated samples, the bacterial community turned out to be significantly site-dependent with the highest alpha diversity found inside the detergent drawer, followed by sump, textile fibres isolated from the washing solution, and door seal. Surprisingly, out of all other investigated factors only the monthly number of wash cycles at temperatures ≥ 60 °C showed a significant influence on the community structure. A higher number of hot wash cycles per month increased microbial diversity, especially inside the detergent drawer. Potential reasons and the hygienic relevance of this finding need to be assessed in future studies.

## 1. Introduction

Today, washing machines are one of the most common household tools targeting household hygiene. The German Federal Statistical Office determined that 96% of German households own a washing machine [1]. Due to this wide distribution, laundering clothes is one of the most widespread housework [2].

The cleaning efficiency of a washing machine is depending on the mechanical circulation of the washing drum, the flooding of the fabric with water and detergents, as well as an appropriate washing time and water temperature [3,4,5]. Mainly for environmental reasons, the washing process has been adapted to sustain energy in order to conserve resources and reduce costs [6]. Sustainable washing trends include washing at lower temperatures, a reduced water consumption and an increased use of bleach-free liquid detergent [7]. However, from a hygienic point of view, these adjustments negatively affect laundry hygiene by facilitating the survival of microorganisms inside the washing machine and on the washed laundry [8].

Recent studies dealing with the antimicrobial effectiveness of modern washing processes showed that microorganism, which mainly enter the machine through worn clothing or water, were reduced, but not sufficiently killed during low temperature wash cycles [8,9]. Surviving microorganism remain inside the washing machine and either attach to different kinds of surfaces or get distributed over the wash load during the wash cycle [10]. The latter might pose a potential risk of infection to members of the household, if pathogens are involved, such as dermatophyte fungi, *Staphylococcus aureus* or *Escherichia coli* [11].

Bacteria bound to surfaces are commonly surrounded by polymeric substances, which is a main characteristic of a biofilm [12]. Biofilms can be formed on almost every surface in an aqueous environment [13]. The formation of biofilms increases microbial tolerance against mechanical, physical and chemical stress [14]. For example, it allows a protected growth, ensures a reduced diffusion rate of toxic components and strengthens the attachment and expansion capabilities of the existing community [14,15]. In addition, the occurrence of many different species at a given site might increase interspecies communication and cross-feeding and positively affect biofilm biomass [16,17]. For the consumer, however, washing machine biofilms are unpleasant, as they are causing a constant recontamination of laundry and regularly cleaned machine parts, malodour of machine and laundry, the formation of unaesthetic plaques and contribute to the corrosion of metallic components [7,9,18]. Finally, they might represent a reservoir for pathogens [19].

In order to characterize the microbial colonization of domestic washing machines, several studies analysed the microbial community of household washing machines and its potential influence on laundry [5,9,20,21,22,23,24,25,26]. For instance, Nix and co-workers [23] addressed the prevalence of prokaryotic and eukaryotic microorganisms at the rubber door seal and the detergent drawer using pyrosequencing of ribosomal RNA and ITS genes. The influence of environmental factors on microbial diversity, however, was not considered here. Stapelton and co-workers [24] investigated potential causes of malodour formation inside the washing machine and their effect on laundry odour. Finally, Callewaert and co-workers [9] showed that bacteria from different sources get significantly mixed during a wash cycle. Interestingly, they also suggested that laundry and washing machine microbial communities might even affect the skin microbiota of their users.

In order to better understand the microbiology of domestic washing machines, our study focused on the influence of selected environmental factors on the bacterial community composition of these widely used items. We hypothesized that factors, such as sampling site, significantly shape community composition. In order to test this hypothesis, we analysed the bacterial community at four different sampling sites using 454-pyrosequencing as a cultivation-independent technique and searched for associations of community composition with selected environmental and user-specific factors.

## 2. Material and Methods

### 2.1. Sample Collection

In the course of this study, 21 in-use domestic washing machines were examined, stemming from private households, either in the area of Villingen-Schwenningen or Waldshut-Tiengen, Germany. Sterile cotton swabs (Deltalab, Rubí, Spain), premoistened in physiological (0.9%) saline solution, were used for taking surface samples of three sampling sites, i.e., detergent drawer (drawer and chamber), door seal and sump. After sampling, the swaps were transferred into a sterile reaction tube and kept at −20 °C until further analysis. In addition to swab samples, fibres released from a wash load into the washing solution were also examined. To do so, machines were loaded with worn cotton laundry and a wash cycle was started at 30 °C with ca. 30 mL of liquid detergent (Persil Universalgel Henkel, Düsseldorf, Germany). After half of the washing cycle (ca. 30 min), the machine was stopped, and washing solution was collected into a sterile 50 mL reaction tube and stored at −20 °C until further processing.

### 2.2. Factors that may Influence Bacterial Diversity in Washing Machines

A survey was issued to the machine owners to gain more information about potential environmental factors affecting the bacterial community composition. Consumers voluntarily and anonymously provided information about the age of the machine, the average number of washing cycles per month at ≥60 °C, the perception of malodour from the machine or washed textiles, as well as the use of fabric softener and the use of powder or liquid detergent. We also asked if a regular cleaning of the machine was done. To simplify the data, factors yielding a wide range of information, such as the age of the machine or the number of wash cycles per month at ≥60 °C were grouped into two categories, each.

### 2.3. DNA-Extraction

Textile fibres from washing solution samples were collected by centrifugation for 5 min at 121× g. Afterwards, the supernatant was discarded, and the pellet was resuspended in 500 μL of PCR-water (Sigma-Aldrich, Hamburg, Germany). DNA from the swap heads and textile fibres was isolated using the FastDNA Spin Kit for Soil and a FastPrep Instrument (both from MP Biomedicals, Eschwege, Germany) using an adjusted protocol including a reduced centrifugation force of 12,100× *g* using a MiniSpin centrifuge (Eppendorf, Hamburg, Germany), as well as an additional protein precipitation step inserted immediately after the one described in the protocol. At the end, the genomic DNA was eluted in 100 μL of DNase/ Pyrogen-free water. Extractions from blank swabs did not yield sufficient DNA for downstream analyses.

### 2.4. PCR and Clean Up

To determine the bacterial community composition, amplicon sequencing based on the 16S rRNA gene was applied. Barcoded amplicons were prepared using universal bacterial primers extended with the respective A or B adapters, a key sequence and a multiplex identifier (MID) sequence [27]. Pyrotag PCR was done using modified ba27f (5′-CGT ATC GCC TCC CTC GCG CCA TCA TCA-MID-Sequence-GAG TTT GAT CMT GGC TCA G-3′) and ba519r (5′-CTA TGC GCC TTG CCA GCC CGC TCA-MID-Sequence-ATT ACC GCG GCT G-3′) primers (Metabion international AG, Martinsried, Germany). Final concentrations for PCR amplification mix were 1× Dream-*Taq*-reaction buffer, 2 mM magnesium chloride, 0.2 mM PCR-nucleotide mix, 1.25 U Dream-*Taq*-polymerase (all from Thermo Fisher Scientific, Waltham, USA), 0.2 µg/µL bovine serum albumin (Roche, Penzberg, Germany), 0.5 µM of each primer and 2 µL DNA template in a final volume of 50 µL. The DNA was amplified using a T-personal thermocycler (Biometra, Göttingen, Germany) with the following thermal profile: 95 °C for 4 min for initial denaturation, followed by 28 cycles of denaturation for 30 s at 94 °C, annealing for 30 s at 52 °C and elongation for 60 s at 72 °C followed by a final elongation for 5 min at 72 °C. Correct amplicon size was verified by gel electrophoresis on 1% agarose gels and ethidium bromide staining. PCR products were purified using the NucleoSpin Gel and PCR Clean up kit (Macherey–Nagel, Düren, Germany) with the MiniSpin centrifuge (Eppendorf, Hamburg, Germany) according to the manufacturer’s protocol, using Tris-HCl-buffer (5 mM, pH 8.5) for elution of DNA. Amplicon concentration and purity were measured with a NanoPhotometer P360 (Implen, München, Germany).

### 2.5. Pyrosequencing

16S rRNA genes amplicons from 50 samples stemming from 13 different machines were sequenced. From one machine only the door seal and detergent drawer yielded sufficient amplicons. The selected amplicon samples were delivered to Eurofins MWG Operon (Ebersberg, Germany) for 454-pyrosequencing using the GS Junior System and the Titanium sequencing kit (both from Roche, Mannheim, Germany).

### 2.6. Bioinformatic and Statistical Analyses

The obtained pyrosequencing data were analysed using QIIME version 1.9.1 [28]. First, the sequences were quality filtered using a quality threshold of 25. Then, the sequences were assigned to their respective samples according to their unique barcode sequence. Reads from forward and reverse primer were merged into one data set and chimeric sequences were removed using the VSEARCH method against the SILVA data base (release SILVA_128_QIIME_release) [29,30]. The remaining sequences were clustered de novo using UCLUST [31] into operational taxonomic units (OTUs) at 97% sequence similarity threshold. Representative sequences were aligned with PyNAST [32] and taxonomy was assigned using the SILVA data base. OTUs from plastids and mitochondria were subsequently removed from the data set. Further processing of the data was done using R version 3.5.3 [33] and RStudio version 1.1.463 [34] with the phyloseq package version 1.26.1 [35] and its additional packages, especially vegan (version 2.5.4) [36]. In a pre-processing step of data analysis, singletons were removed from the data set, followed by rarefaction to the minimal sequence count of all samples. In order to describe the microbial community composition, the overall relative abundance as well as the relative abundance of the OTUs at the respective sampling sites was calculated. The four most common indices (Observed, Chao1, Shannon and Simpson) were used to determine alpha diversity. A subsequent Analysis of Variance (ANOVA) was used for statistical analysis of the influence of the recorded factors on alpha diversity. Differences in beta diversity were visualised by principal component analysis (PCoA) of weighted and unweighted UniFrac measures. We used Analysis of Similarities (ANOSIM) and Permutational Multivariate Analysis of Variance (PERMANOVA) with 9999 permutations to check whether samples show statistically significant differences in community structure at the different sampling sites. Kruskal-Wallis analysis was done to analyse significant differences in bacterial community composition at the different sampling sites. Using the Wilcoxon-Mann-Whitney-U test for independent samples, we investigated the influence of the number of wash cycles per month ≥60 °C on community composition for each sampling site. *p*-values were adjusted for multiple testing by calculating the False Discovery Rate (FDR) using the Benjamini and Hochberg method [37]. *p*-values < 0.05 were regarded as statistically significant. Data were visualized using ggplot2 (version 3.1.0) [38]. To further identify the ten relatively most abundant OTUs per sampling site at species level, we performed a pairwise alignment using EzBioCloud (https://www.ezbiocloud.net/) [39] against a database of 16S rRNA gene sequences (EzBioCloud App: 16S-based ID, September 2019). Identified OTUs were classified into risk groups according to the German Technical Rules for Biological Agents (TRBA) #466 [40]. All sequence data were deposited at the European Nucleotide Archive (ENA) under the accession number PRJEB35498.

## 3. Results

### 3.1. General Bacterial Community Composition

454-pyrosequencing of the 16S rRNA gene amplicon library resulted in a total number of 110,751 raw sequences from the 50 samples, stemming from 13 domestic washing machines. After length/quality filtering, a total of 57,563 high quality forward reads and 44,564 high quality reverse reads were received. These data sets were combined and chimeric sequences (18,042) were removed. The remaining 81,206 sequences were then clustered de novo into 9211 OTUs that shared a 97% sequence similarity threshold. Further removal of mitochondrial and chloroplastic OTUs yielded 7080 bacterial OTUs, representing a total of 77,996 high quality sequences with 353–6802 sequences per sample (mean of 1560 reads per sample). After removal of singletons (4150), the whole data set was rarefied to 242 sequences per sample. Finally, 16 phyla, 36 classes, 67 orders, 124 families, 214 genera and 229 species-like OTUs were determined as components of the bacterial community inside the investigated washing machines.

At phylum level, Proteobacteria (85.8%) was by far the dominating phylum, followed by Actinobacteria (5.3%), Firmicutes (3.0%), Bacteroidetes (2.9%) and Acidobacteria (1.1%). At class level, most sequences were affiliated with Gammaproteobacteria (57.8%), followed by Alphaproteobacteria (17.5%) and Betaproteobacteria (10.3%). Further common classes inside the washing machines were Actinobacteria (5.2%), Bacilli (2.7%), Flavobacteria (2.2%) and Blastocatellia (1.1%), whereas the main identified orders were Pseudomonadales (50.9%), Rhizobiales (9.8%) and Burkholderiales (8.3%). Within the family level, most bacteria belonged to Pseudomonadaceae (30.9%), Moraxellaceae (21.5%), and Comamonadaceae (7.0%). The predominant genera could be identified as Pseudomonas (34.3%), Acinetobacter (17.4%) and Enhydrobacter (6.5%).

### 3.2. Site-Dependent Bacterial Community Composition

Differences in bacterial diversity were investigated by alpha diversity using observed OTUs, Chao1, Shannon and Simpson as parameters (Table 1).

All diversity indices showed significant differences across the sampling sites (ANOVA: *p*_Observed_ = 6.5 × 10^−4^, *p*_Chao1_ = 5.6 × 10^−3^, *p*_Shannon_ = 9.3 × 10^−4^, *p*_Simpson_ = 3.8 × 10^−3^). The highest alpha diversity was found for the detergent drawer, followed by the fibres isolated from the washing solution, and the sump. The lowest alpha diversity was found inside the door seal.

**Figure 1:**
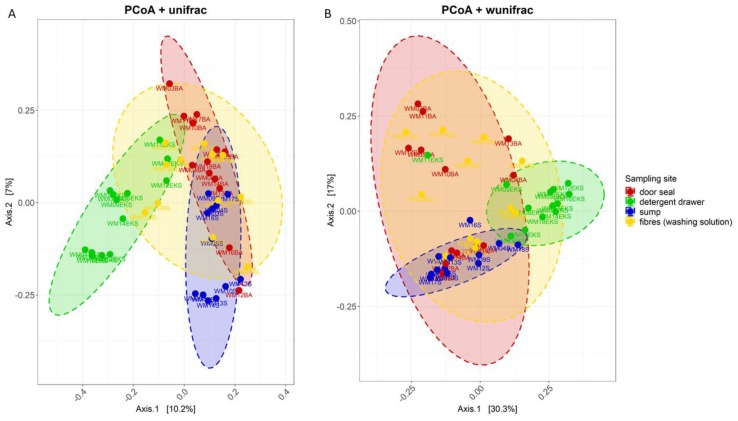
Principal Coordinate Analysis (PCoA) plot of unweighted (A) and weighted (B) UniFrac measures using the 16S rRNA gene sequencing data of 50 analysed washing machine samples. Colour indicates sampling site: door seal (red), detergent drawer (green), sump (blue) and fibres from washing solution (yellow). Ellipses correspond to 95% confidence intervals for each of the four sampling sites.

In order to visualize differences in community structure between the different sampling sites, principal component analysis using weighted und unweighted Unifrac measures was done (Figure 1). Samples that originated from the detergent drawer were clearly distinct from the sump, which in turn were different from the door seal or the fibre samples. A segregation of the samples from door seal and the fibres becomes visible at the unweighted Unifrac distances, whereas the weighted analysis showed an overlay. The statistical analysis by means of PERMANOVA (*p* = 1 × 10^−4^ for unweighted Unifrac and weighted Unifrac) and ANOSIM (unweighted Unifrac: R = 0.4; weighted Unifrac: *R* = 0.3, *p* = 1 × 10^−4^ for unweighted Unifrac and weighted Unifrac) showed that the structure of the bacterial community at the sampling sites was significantly different.

**Figure 2:**
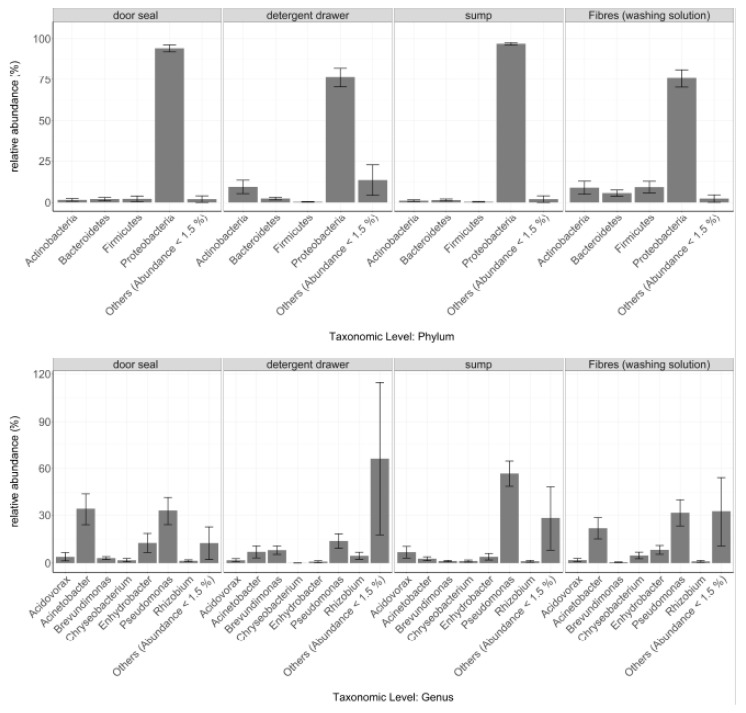
Relative abundances of the most abundant microbial phyla and genera at the different sampling sites. Only taxa with an overall relative abundance of ≥ 1.5% are shown Taxa with an overall relative abundance ≤ 1.5% were summed up as “Others”. Data are expressed as mean ± standard error (Door seal *n* = 13, Detergent drawer *n* = 13, Sump *n* = 12, Washing solution *n* = 12).

Consequently, also the distribution of the different taxa was found to be highly site-dependent (Figure 2), in particular the phylum of Proteobacteria (Kruskal-Wallis: *p* = 8.1 × 10^−4^). Proteobacteria were found across all sampling sites, but in case of the door seal and the sump, this phylum accounted for 94.2% and 96.9% of all sequences, respectively, while the proportion in the detergent drawer (76.3%) and on the fibres from the washing solution (75.8%) was significantly lower. Firmicutes (Kruskal- Wallis: *p* = 8.8 × 10^−3^), however, were mainly found on the fibres isolated from the washing solution (9.3%) and in the door seal (2.2%). Furthermore, the relative abundance of Actinobacteria also depended strongly on the sampling site (Kruskal-Wallis: *p* = 0.02). Here, we found frequencies of around one to two percent in the sump and the door seal. The washing solution fibres and the detergent drawer on the other hand showed relative abundances of ~9%. In addition to the most common phyla, other phyla also showed significant differences between sampling sites. For instance, the phyla Planctomycetes (Kruskal-Wallis: *p* = 8.1 × 10^−4^), Chloroflexi, (Kruskal-Wallis: *p* = 8.8 × 10^−3^) and Acidobacteria (Kruskal-Wallis: *p* = 3.7 × 10^−3^) were found mainly in the detergent drawer but rarely at the other sampling sites.

At genus level, the genera Pseudomonas (Kruskal-Wallis: *p* = 9.8 × 10^−3^), Acinetobacter (Kruskal-Wallis: *p* = 0.01) and Enhydrobacter (Kruskal-Wallis: *p* = 0.03) were found at all sampling sites. However, their relative abundances varied greatly. For instance, the relative abundance of Pseudomonas in the detergent drawer (13.7%) was much lower compared to the sump (56.7 %). On the other hand, the relative abundance of this genus for door seal (32.9%) and fibres (31.4%) was similar. In contrast, Enhydrobacter occurred mostly in the door seal (12.5%) and on the textile fibres (8.2%) but only barely in the detergent drawer (0.8%). Acinetobacter, in turn, occurred more often in the door seal (34.0%), followed by the washing solution fibres (21.7%). Its relative abundance, however, was significantly lower in the detergent drawer (6.9%) and the sump (2.7%).

In order to further identify the ten most abundant OTUs per site at species level, we calculated sequence similarity against the 16S rRNA gene sequences database from EzBioCloud (Table A1). Notably, this analysis clearly revealed that the OTUs previously identified as Enhydrobacter showed a sequence similarity of 100% to the species *Moraxella osloensis*.

Significant fractions (30–60%) of the 10 relatively most abundant OTUs per sampling site could be categorized as closely related to potentially pathogenic species based on the German TRBA #466, and many of these OTUs were detected at the majority of the investigated sites. For instance, OTUs closely related to *Moraxella osloensis* were detected in up to 6 sump, 8 fibre and 9 door seal samples (Table A1).

### 3.3. Effect of Environmental Factors on Community Composition

**Figure 3:**
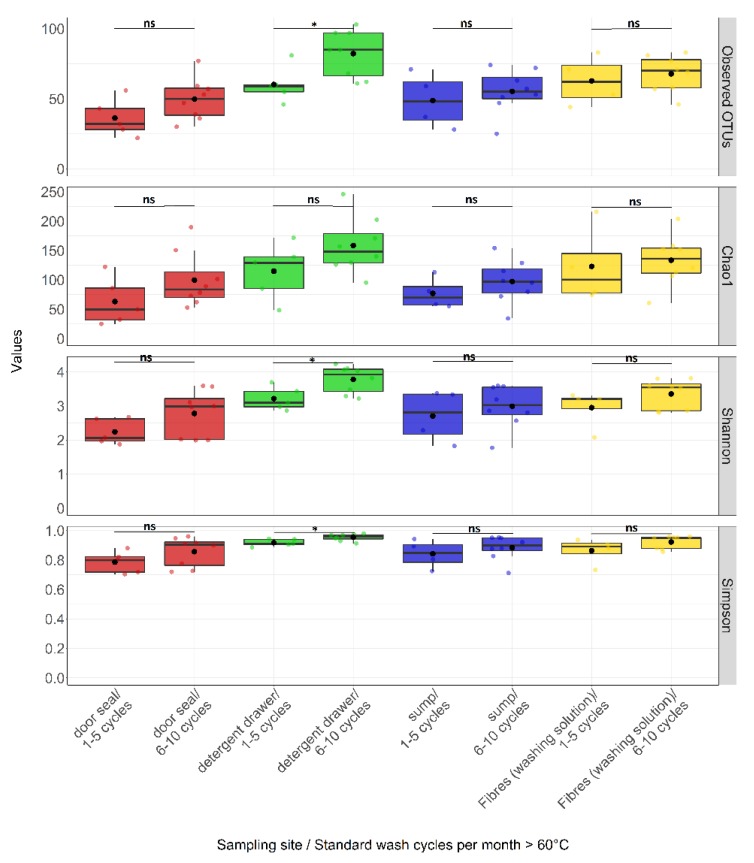
Box-whisker plots for the distribution of alpha diversity measures comparing sampling sites by standard wash cycle per month at temperatures ≥60 °C. Samples were categorized by sampling sites and the number of standard wash cycle per month at temperatures ≥60 °C. A box represent the 25% and 75% percentiles. The middle of the box represents the median. Mean values are displayed as black dots. The horizontal dashes above and below the boxes indicate the largest and smallest values, which were not classified as outliers. Colour indicates sampling site: door seal (red), detergent drawer (green), sump (blue) and fibres from washing solution (yellow). Coloured dots represent an individual sample. Statistical analysis was done using the Wilcoxon-Mann-Whitney-U tests for independent samples. *p*-values are indicated by asterisks, *p* < 0.05 (*) and ns = not significant.

In addition to the clear effects of sampling site on bacterial community composition, we investigated the effects of further parameters with a potential influence on microbial community composition. Unexpectedly, the performed ANOVA analysis revealed that only the number of wash cycles per month at ≥60 °C seemed to have an impact on the microbial diversity (*p*_Observerd_ = 0.04, *p*_Chao1_ = 0.06, *p*_Shannon_ = 0.04, *p*_Simpson_ = 0.04). Surprisingly, there was a trend towards a higher alpha diversity with an increased number of wash cycles ≥60 °C compared to a lower number of wash cycles at high temperature (Table 1). Furthermore, we also examined at which sampling site this factor had the strongest effect on microbial diversity. Figure 3 shows that there was a significantly higher alpha diversity in the detergent drawer from machines which undergo 6–10 washing cycles per month at ≥60 °C. At the other sampling sites, no clear influence of this parameter was seen.

Beta diversity revealed no clear differences between a higher and lower number of wash cycles at temperatures above ≥60 °C using PCoA or ANOSIM and PERMANOVA (data not shown). We therefore compared the relative abundances of single taxa between a high and a low number of wash cycles above 60 °C. A significant difference between a high and low numbers of wash cycles ≥60 °C was seen for the order of Xanthomonadales (Wilcoxon: *p* = 7.3 × 10^−3^). Its relative abundance increased with a higher number of wash cycles ≥60 °C from 0.6% to 4.8%. At the genus level, a borderline significant difference was determined for Paracoccus (Wilcoxon: *p* = 0.05). Its relative abundance increased from 0.2% at 1–5-wash cycles to 1.8% at 6–10 high-temperature wash cycles per month. Also, the minor abundant genera Kocuria, Dysgonomonas, Massilia (Wilcoxon: each *p* = 0.03) differed significantly between these two conditions.

## 4. Discussion

One of the main objectives of our study was to identify potential factors influencing microbial diversity in domestic washing machine. In order to achieve this, we examined 13 different household washing machines at four different sampling sites by means of 454-pyrosequencing for their bacterial community composition and statistically analysed associations with different environmental and user specific factors.

### 4.1. The structure of the Bacterial Community Differs between Various Sampling Sites

Using different alpha diversity parameters, we compared the specific sampling sites and were able to determine the highest alpha diversity with a high evenness for the detergent drawer and the lowest diversity for the door seal with a relatively low evenness, which corroborates findings by Nix and colleagues [23]. Similar to our study, they also identified Proteobacteria, Actinobacteria and Bacteroidetes as the main phyla in washing machines. However, we additionally examined the sump and fibres collected from the washing solution, which extends knowledge of the core microbiome in washing machines. Firmicutes were relatively most abundant on textile fibres from the washing solution, which seems likely as Firmicutes are typical representatives of the human skin microbiota, such as staphylococci [41,42]. Diversity and species richness of sump and fibres were between the values determined for detergent drawer and door seal.

Local conditions probably play a decisive role for bacterial community composition in washing machines and may select for polyextremotolerant bacteria [43]. For example, bacteria in the detergent drawer must be particularly tolerant to the ingredients of the detergent, such as bleach, surfactants, perfumes or enzymes, and alkaline components [43,44]. In contrast, bacteria within the door seal need to handle high organic loads caused by the washed objects, alternating phases of dry and very wet conditions and changing pH values [21].

The relative abundances clearly show that some bacteria are restricted to certain sites, such as the aforementioned phylum of Firmicutes. In contrast, Proteobacteria were found in all analyzed sampling sites. Proteobacteria are known to be the most common bacteria in drinking water [45,46] and tap water serves as a means of transportation throughout all components of the machine. Accordingly, the genus Pseudomonas, also very typical for drinking water [47,48], was found at all sampling sites.

Genera such as Moraxella and Acinetobacter are members of the human skin microbiome and probably enter the machine mostly with dirty laundry [49,50]. Since the water flow in the machine is unidirectional and the washing solution does not come into contact with the detergent drawer, these bacteria were only rarely detected there. Our analyses down to species level suggest that the different sampling sites of a washing machines are not only populated by harmless environmental bacteria, but also by potentially pathogenic ones, in particular *Acinetobacter spp.* and *Moraxella osloensis*. For healthy people these bacteria are rather harmless. However, in new-borns, pregnant women, elderly persons or other immunocompromised subjects they might lead to infections [51,52,53].

*Moraxella osloensis* was also identified as a cause of malodor on laundry [8,24,54]. In particular *Moraxella osloensis’s* ability to tolerate desiccation and a metabolic pathway to produce 4-methyl-3-hexenoic acid are considered key factors for survival and malodor formation in washing machines and on washed laundry [55]. Using a detergent containing disinfecting agents or bleach is recommended to control malodor formation [54]. In addition, machine parts in direct contact with laundry, that are prone to the growth of malodor producers, should be cleaned on a regular basis. In our study, an OTU closely related to *Moraxella osloensis* (sequence similarity: 100%) was relatively most abundant on the door seals. Hence regular cleaning here appears particularly suited to control this bacterium and its ability to produce malodour. Unfortunately, quantitative data on the bacterial colonization of washing machines is scarce, and in our study only relative abundances of different taxa were determined. However, in a small study Stapleton and colleagues [24] clearly showed that bacterial cell numbers of sump and rubber seal were several log scales higher than cell numbers of washing drum and drawer. Therefore, it can be carefully speculated that the prominent occurrence of *Moraxella osloensis* in the door seal samples of our study is indeed of quantitative relevance.

Finally, it is noteworthy that during the OTU identification down to species level a misclassification was revealed. OTUs classified by QIIME as Enhydrobacter were clearly identified as Moraxella, more precisely *Moraxella osloensis,* by pairwise alignment to the EzBioCloud database. The same misclassification was demonstrated before [56].

### 4.2. Factors Influencing Bacterial Community Composition

In addition to the influence of the sampling sites, we also investigated other, user-dependent factors. Unexpectedly, only the number of wash cycles at ≥60 °C significantly influenced the community composition, while factors such as the age of the machine or regular cleaning measures did not, at least when based on our data set.

When comparing machines with different number of washing cycles per month at temperatures ≥60 °C, an effect on alpha diversity, but not on beta diversity was seen. Different alpha diversity parameters revealed a tendency towards an increase in bacterial diversity at 6–10 washing cycles per month at ≥60 °C compared to a number of 1–5 washing cycles. This effect was only observed for the detergent drawer, but not for sites with direct contact to the heated water. Therefore, this positive effect on bacterial diversity might be caused by heat radiation from the washing drum to the surrounding components, where it might stimulate microbial growth. However, it cannot be excluded that other factors, not recorded in this study, were responsible for this observation, such as the general number of washing cycles at both low and high levels. It might be speculated that households with a higher number of washing cycles at 60 °C or higher per month also perform more washings at lower temperatures, which in turn may influence microbial diversity.

## 5. Conclusions

Domestic washing machines are colonized by a diverse bacterial community probably affecting laundry hygiene. The bacterial community is dominated by taxa of water and human origin. Bacterial diversity is strongly site-dependent and shaped by the local environmental conditions. Some of the identified bacterial species here are categorized as potentially pathogenic species, that might be transmitted through laundry and cause infections in immunocompromised humans. We also demonstrated that the bacterial community composition in the detergent drawer might be influenced by the number of wash cycles per month at temperatures ≥ 60°C. Potential reasons and the hygienic relevance of this finding need to be assessed in future studies.

Clearly, such follow-up studies should not only be based on relative amplicon frequencies of 16S rRNA genes but include both quantitative (cultivation-based cell numbers, qPCR-based gene numbers) as well as more functional oriented (transcriptomic, proteomic, metabolomic) analyses in order to better understand the microbiology of domestic washing machines and its hygienic relevance.

## Figures and Tables

**Table 1:** Summary of the distribution of alpha diversity indices for the investigated factors potentially influencing the bacterial community of washing machines. Factors that had a significant influence are highlighted in grey. Displayed are mean values, minimum and maximum value as well as standard deviation of each diversity index.

Influencing Factor	Levels	n	Observed				Chao1				Shannon				Simpson			
			Mean	SD	Min	Max	Mean	SD	Min	Max	Mean	SD	Min	Max	Mean	SD	Min	Max
**Site**	Door seal	13	44.54	15.50	22.00	77.00	85.44	46.88	25.00	189.80	2.57	0.63	1.88	3.59	0.83	0.09	0.70	0.96
	Detergent drawer	13	73.77	18.49	46.00	103.00	141.64	51.03	48.15	246.00	3.56	0.46	2.87	4.24	0.94	0.03	0.89	0.98
	Sump	12	53.08	16.47	25.00	74.00	90.37	33.98	34.17	154.09	2.89	0.66	1.77	3.59	0.87	0.09	0.71	0.95
	Fibres (washing solution)	12	66.08	14.08	44.00	83.00	129.83	48.74	60.62	216.00	3.22	0.50	2.08	3.81	0.90	0.07	0.73	0.96
**Age**	0 - 10 years	34	58.85	20.96	22.00	103.00	113.79	55.82	25.00	246.00	3.03	0.73	1.83	4.24	0.88	0.09	0.70	0.98
	11 -20 years	16	60.44	16.74	25.00	97.00	107.85	39.38	34.17	171.88	3.14	0.53	1.77	4.07	0.90	0.06	0.71	0.97
**Smell**	Yes	10	62.44	20.70	28.00	97.00	123.02	54.57	32.00	216.00	3.11	0.72	1.83	4.24	0.89	0.08	0.72	0.98
	No	40	58.80	17.31	32.00	97.00	105.52	40.28	49.50	156.91	3.13	0.58	1.97	4.07	0.90	0.08	0.70	0.97
**Standard wash cycles per month ≥60 °C**	1 - 5 cycles	18	51.56	18.10	22.00	83.00	93.69	50.01	25.00	216.00	2.77	0.61	1.83	3.69	0.85	0.08	0.70	0.94
	6 -10 cycles	32	63.75	19.21	25.00	103.00	122.13	49.07	34.17	246.00	3.23	0.65	1.77	4.24	0.90	0.08	0.71	0.98
**Regular Cleaning**	Yes	20	62.95	20.69	25.00	97.00	118.60	49.36	32.00	216.00	3.14	0.70	1.77	4.24	0.89	0.08	0.71	0.98
	No	30	56.97	18.72	22.00	103.00	107.41	52.09	25.00	246.00	3.01	0.65	1.97	4.10	0.88	0.08	0.70	0.97
**Softener**	Yes	16	62.44	20.70	28.00	97.00	123.02	54.57	32.00	216.00	3.11	0.72	1.83	4.24	0.89	0.08	0.72	0.98
	No	34	57.91	19.12	22.00	103.00	106.65	48.89	25.00	246.00	3.04	0.65	1.77	4.10	0.88	0.08	0.70	0.97
**Detergent**	Liquid	18	57.78	13.61	32.00	85.00	109.06	37.93	49.50	171.88	3.03	0.54	1.97	4.03	0.89	0.08	0.70	0.97
	Powder	32	60.25	22.36	22.00	103.00	113.48	57.30	25.00	246.00	3.08	0.74	1.77	4.24	0.88	0.09	0.71	0.98

## Appendix A

**Table A1:** Ten relatively most abundant OTUs per sampling site. The ten relatively most abundant OTUs per sampling site were determined and aligned against the 16S rRNA gene sequence database of EzBioCloud to calculate sequence similarities to known species. For each EzBioCloud match, sequence similarity and completness values are displayed. The identified top-hit taxa were also categorized into risk groups (RG) according to the German TRBA 466 [40]. SD = standard deviation. Positive samples = number of samples in which the OTU was detected.

Sampling Site	Relative Abundance (%)	SD	Positive Samples	OTU-ID	SILVA Genus	Top-hit taxon EzBioCloud	EzBioCloud Accession Number	Similarity (%)	Completeness (%)	RG (TRBA)
**Door seal**	12.1	14.8	11	denovo7218	Pseudomonas	*Pseudomonas oleovorans subsp. oleovorans*	NIUB01000072	100.00	33.3	1
	10.1	13.3	11	denovo5159	Pseudomonas	*Pseudomonas oleovorans subsp. oleovorans*	NIUB01000072	100.00	33.2	1
	7.2	14.6	8	denovo7377	Acinetobacter	JF232448_s	JF232448	98.97	33.3	-
	6.0	11.2	9	denovo6937	Enhydrobacter	*Moraxella osloensis*	APQL01000005	100.00	33.6	2
	4.1	13.5	2	denovo3157	Acinetobacter	*Acinetobacter beijerinckii*	APQL01000005	99.38	33.3	2
	3.8	6.7	7	denovo301	Enhydrobacter	*Moraxella osloensis*	CP014234	100.00	33.1	2
	3.6	8.3	7	denovo6136	Acinetobacter	*Acinetobacter parvus*	AIEB01000124	100.00	33.4	2
	3.4	8.9	5	denovo4753	Acinetobacter	*Acinetobacter parvus*	AIEB01000124	100.00	33.3	2
	3.1	7.3	5	denovo3836	Acidovorax	*Acidovorax radicis*	AFBG01000030	99.59	33.2	1
	2.7	5.9	7	denovo3896	-	*Rhizobium rosettiformans*	EU781656	98.91	32.6	1
**Detergent drawer**	2.5	6.6	4	denovo7377	Acinetobacter	JF232448_s	JF232448	98.97	33.3	-
	2.3	4.3	10	denovo15	Brevundimonas	*Brevundimonas vesicularis*	BCWM01000033	98.48	33.5	2
	2.1	4.5	4	denovo8752	-	*Pseudoxanthomonas mexicana*	AF273082	100.00	33.5	1
	1.9	7.0	1	denovo6661	uncultured	HQ856368_s	HQ856368	100.00	32.4	-
	1.9	5.3	3	denovo1071	Pseudomonas	*Pseudomonas aeruginosa*	BAMA01000316	99.00	34.2	2
	1.9	5.7	6	denovo5179	-	*Pseudoxanthomonas mexicana*	AF273082	99.20	34.6	1
	1.7	3.9	5	denovo7389	Rhizobium	*Rhizobium radiobacter*	AJ389904	98.47	32.8	1
	1.5	3.3	7	denovo2452	Brevundimonas	*Brevundimonas vesicularis*	BCWM01000033	100.00	31.4	2
	1.4	3.1	4	denovo8373	Aureimonas	*Aureimonas altamirensis*	BBWQ01000019	98.26	33.0	1
	1.4	4.9	1	denovo5170	Pseudomonas	*Pseudomonas avellanae*	AKBS01001374	100.00	33.4	1
**Sump**	20.1	14.0	12	denovo7218	Pseudomonas	*Pseudomonas oleovorans subsp. oleovorans*	NIUB01000072	100.00	33.3	1
	17.0	11.8	12	denovo5159	Pseudomonas	*Pseudomonas oleovorans subsp. oleovorans*	NIUB01000072	100.00	33.2	1
	4.7	9.0	5	denovo3836	Acidovorax	*Acidovorax radicis*	AFBG01000030	99.59	33.2	1
	2.6	5.0	5	denovo2699	Citrobacter	*Citrobacter freundii*	AJ233408	99.80	33.5	2
	2.3	3.0	8	denovo4149	-	*Diaphorobacter nitroreducens*	AB064317	99.59	33.3	1
	1.5	2.4	5	denovo7180	-	*Diaphorobacter nitroreducens*	AB064317	97.96	34.2	1
	1.5	2.9	6	denovo6937	Enhydrobacter	*Moraxella osloensis*	CP014234	100.00	33.6	2
	1.4	4.1	5	denovo213	Ochrobactrum	*Ochrobactrum anthropi*	CP000758	100.00	30.7	2
	1.2	2.6	5	denovo301	Enhydrobacter	*Moraxella osloensis*	CP014234	100.00	33.1	2
	1.2	2.0	4	denovo6665	-	*Kosakonia sacchari*	CP007215	99.80	33.4	-
**Fibres (Washing solution)**	10.7	12.7	9	denovo7218	Pseudomonas	*Pseudomonas oleovorans subsp. oleovorans*	NIUB01000072	100.00	33.3	1
	10.3	12.4	10	denovo5159	Pseudomonas	*Pseudomonas oleovorans subsp. oleovorans*	NIUB01000072	100.00	33.2	1
	5.4	9.3	8	denovo7377	Acinetobacter	JF232448_s	JF232448	98.97	33.3	-
	3.0	3.9	9	denovo301	Enhydrobacter	*Moraxella osloensis*	CP014234	100.00	33.1	2
	2.9	4.9	5	denovo7076	Micrococcus	*Micrococcus aloeverae*	KF524364	100.00	32.3	-
	2.4	3.4	9	denovo2637	Acinetobacter	*Acinetobacter johnsonii*	APON01000005	98.97	33.4	2
	2.4	2.6	8	denovo6937	Enhydrobacter	*Moraxella osloensis*	CP014234	100.00	33.6	2
	2.1	3.7	6	denovo1609	Chryseobacterium	*Chryseobacterium hominis*	jgi.1096633	99.00	34.7	2
	2.0	6.9	1	denovo5400	Acinetobacter	*Acinetobacter junii*	APPX01000010	98.96	33.0	2
	1.8	2.7	7	denovo2396	Chryseobacterium	*Chryseobacterium hominis*	jgi.1096633	99.79	33.4	2
